# Dataset on heavy metal content in background soils of the three gully catchments at Western Siberia

**DOI:** 10.1016/j.dib.2019.104496

**Published:** 2019-09-11

**Authors:** Ivan Semenkov, Anton Yakushev

**Affiliations:** a Lomonosov Moscow State University, Russia; b Institute of Geology of Ore Deposits, Petrography, Mineralogy and Geochemistry of Russian Academy of Sciences, Russia

**Keywords:** Potentially toxic elements, Retisols, Phaeozems, Chernozems, Soil quality standards, Soil pollution, Environmental policy, Human health risk assessment

## Abstract

The criteria used include heavy metal (HM) levels in background soils of different countries and territories (Australia, China, Finland, North America, Northern Europe, and Western Siberia) and their threshold concentration values for soils of residential and/or agricultural areas in soil quality standards of Canada (soil quality guidelines), Germany (Trigger Values), the Netherlands (Serious Risk Concentrations), Russia (Maximum Permissible Concentrations), and the USA (Regional Screening Levels). The Retisols, Phaeozems and Chernozems of Western Siberia are characterized by the following range of mean concentrations of heavy metals in topsoil (in mg kg^−1^): Pb 5–35, Cu 5–100, V 5–180, Cr 5–212, Ni 7–100, Zn 10–135, Mn 50–1800, and Ba 373–1360.

**Table:** Specifications Table

Subject area	*Earth Planetary Science*
More specific subject area	*Soil Science, Environmental Chemistry*
Type of data	*Tables*
How data was acquired	*Data were obtained by means of standard techniques, an ‘Analizeter 22’ equipment (Germany), an Axios X-Ray fluorescence spectrometer (made by PANalytical, Netherlands)**[1]*. *Data on heavy metal concentration in the upper part of continental earth's crust (UCEC) and background soils of the world were estimated using published materials.*
Data format	*Raw and analysed*
Experimental factors	*Soil samples were taken for heavy metal analysis in the interfluves, slopes and gully bottoms at three gully catchments at Western Siberia*
Experimental features	*The eight heavy metals as stated in the abstract were analysed statistically and compared with abundance of elements within the upper part of the continental earth's crust, their levels in background soils of countries and their threshold concentration values for soils of residential and/or agricultural areas in soil quality standards.*
Data source location	*Key area 1 – the upland of Tobolsky Materik – N 58°56′ E 69°10′* *Key area 2 – the north of the Ishim Plain – N 56°31′ E 67°32′* *Key area 3 – the eastern part of the Trans-Urals region N 56°02′ E 63°35′*
Data accessibility	*All the data are in this article*
Related research article	*I.N.Semenkov, E.V.Terskaya, N.S.Kasimov. Heavy metals' lateral fractionation in catenae of the central part of the Western Siberia. Vestnik Moskovskogo Unviersiteta, Seriya Geografiya, 2019 (3) 25 – 37.*

**Table:** 

**Value of the data** • First open access database on variations of heavy metals content in soils of small catchments at Western Siberia. • These data are valuable for researchers interested in soil geochemistry. Policy makers, government official and stakeholders can benefit from this dataset. This data allows other researchers to extend the statistical analyses. • This dataset can be used for benchmarking studies by comparing parameters of the Western Siberia soils to parameters of the other Retisols, Phaeozems and Chernozems. • The data in this article may be used for educational and technical course purposes. • The data set may be relevant for future researchers about soil geochemistry in the Western Siberia, for example, for ecological modeling.

## 1 Data

According to soil maps [9] of lowland regions of countries within the temperate zone of the northern hemisphere, Russia has close similarities with Canada, Germany, Finland, the Netherlands, Norway, Poland, Sweden and the USA in terms of the occurrence and distribution of reference soil groups (Table 1).

**Table 1:** Reference soil groups at different countries within the temperate zone of the northern hemisphere.

Soils	Russia	the USA	Cabada	Germany	Poland	the Netherlands	Finland	Norway	Sweden	France	the UK	Denmark
1. Soils with thick organic layers:												
Histosols	+++	++	+++	+++	+++	+++	+++	++	++	–	–	++
2. Soils with strong human influence –												
Anthrosols	–	–	–	–	–	–	–	–	–	–	–	–
Technosols	–	–	–	–	–	–	–	–	–	–	–	–
3. Soils with limitations to root growth –												
Cryosols	+++	+++	+++	–	–	–	+	+	+	–	–	–
Leptosols	–	–	–	–	–	–	–	–	–	–	–	–
Solonetz	++	++	–	–	–	–	–	–	–	–	–	–
Vertisols	–	+++	–	–	–	–	–	–	–	–	–	–
Solonchaks	+	++	–	–	–	–	–	–	–	–	–	–
4. Soils distinguished by Fe/Al chemistry –												
Gleysols	+++	+++	+++	+	+	++	–	–	–	+	+++	–
Andosols	+++	+++	+++	–	–	–	–	–	–	–	–	–
Podzols	+++	+++	+++	+++	+++	+	+++	+++	+++	–	–	–
Plinthosols	–	–	–	–	–	–	–	–	–	–	–	–
Nitisols	–	++	–	–	–	–	–	–	–	–	–	–
Ferralsols	–	–	–	–	–	–	–	–	–	–	–	–
Planosols	++	+++	–	–	–	–	–	–	–	+	–	–
Stagnosols	+++	+++	+++	++	++	++	++	++	++	+++	+++	++
5. Pronounced accumulation of organic matter in the mineral topsoil –												
Chernozems	+++	+++	+++	–	–	–	–	–	–	–	–	–
Kastanozems	+++	+++	++	–	–	–	–	–	–	–	–	–
Phaeozems	+++	+++	++	–	–	–	–	–	–	–	–	–
Umbrisols	–	+++	–	+	+	–	+	+	–	++	–	–
6. Accumulation of moderately soluble salts or non–saline substances –												
Durisols	+++	+++	+++	++	+	++	–	–	–	++	+++	++
Gypsisols	–	–	–	–	–	–	–	–	–	–	–	–
Calcisols	–	+++	–	–	–	–	–	–	–	–	–	–
7. Soils with a clay–enriched subsoil –												
Retisols	+++	+++	+++	+++	+++	+++	+++	+++	+++	+++	+++	+++
Acrisols	–	++	–	–	–	–	–	–	–	–	–	–
Lixisols	–	–	–	–	–	–	–	–	–	–	–	–
Alisols	–	++	–	–	–	–	–	–	–	–	–	–
Luvisols	+++	+++	+++	+++	+++	+++	–	–	–	+++	+++	+++
8. Soils with little or no profile differentiation –												
Cambisols	+++	+++	+++	+++	+++	+++	+++	+++	+++	+++	+++	+++
Arenosols	+++	+++	+++	+	+	–	–	–	–	+	+	–
Fluvisols	–	++	–	–	–	–	–	–	–	–	–	–
Regosols	–	++	–	–	–	–	–	–	–	–	–	–

**Fig. 1:**
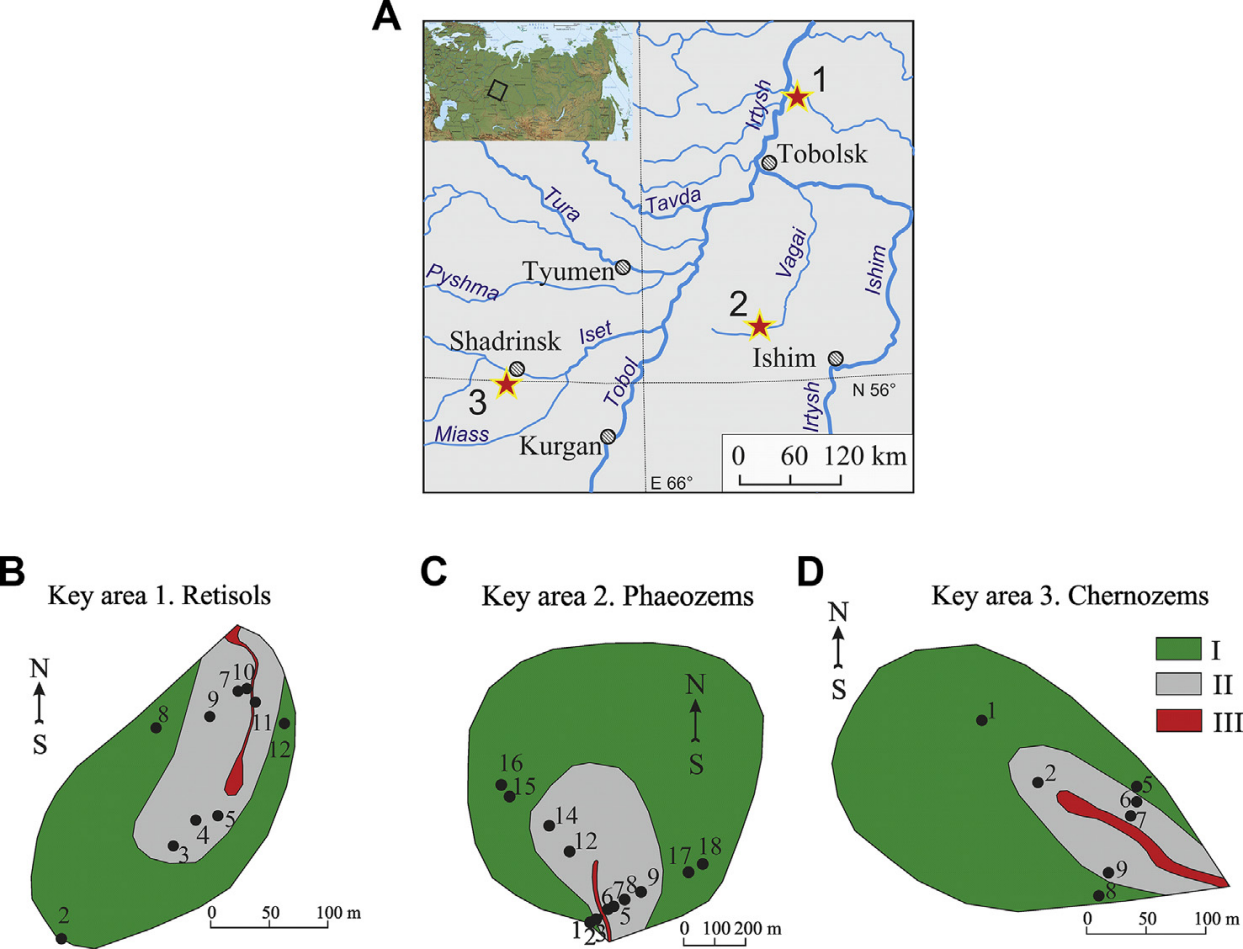

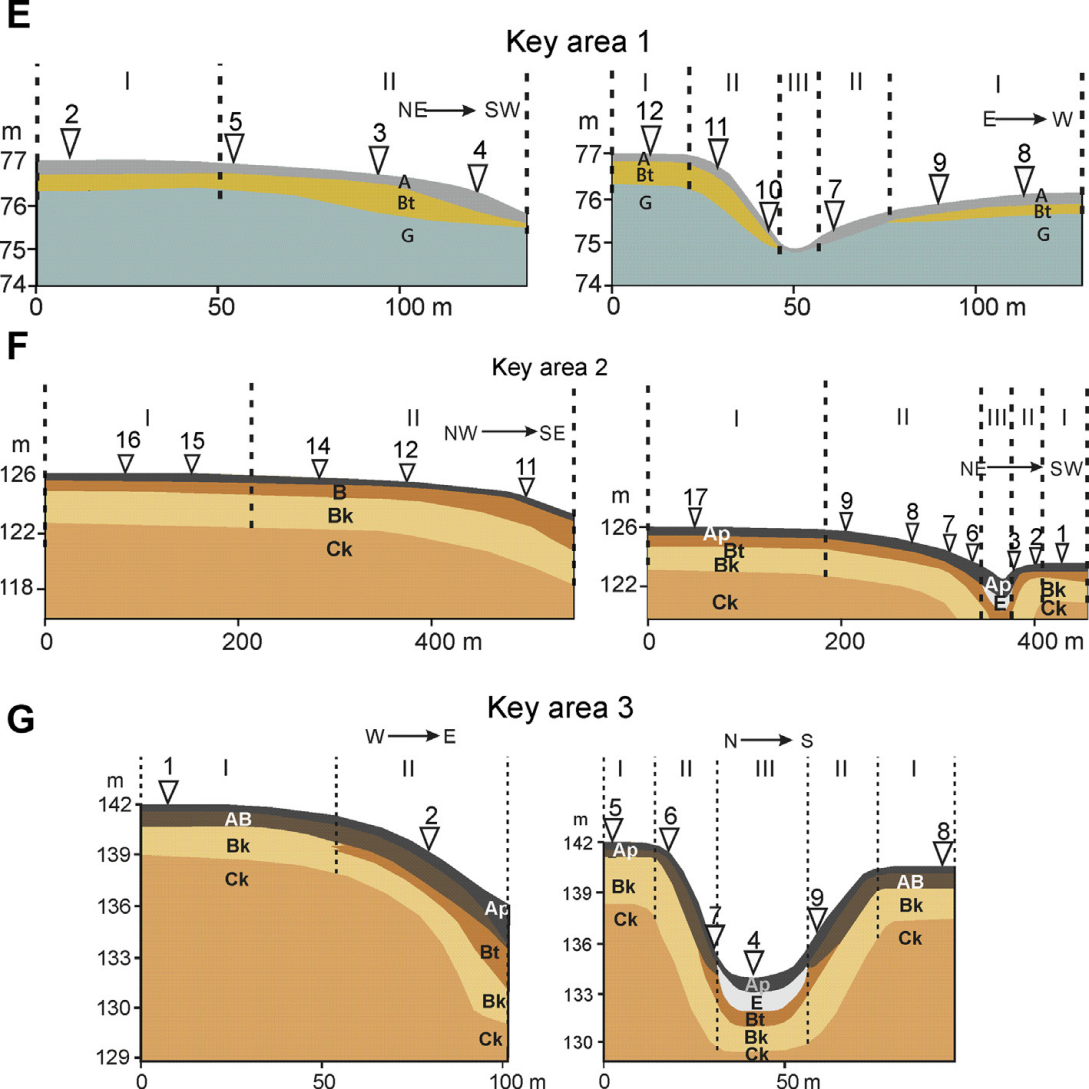
The location of sampling area. Soil pits (numbers 1–18) are located in the following topographic locations: I – the levelled interfluve, II – slopes and III – the bottom of the U-shaped gully.

The field studies on soils were conducted in Western Siberia, at three sites within small gully catchments (Table 2, Fig. 1). The study sites were selected on the basis of analysing the maps of vegetation, soils, parent materials and geochemical migration factors within the Ob river basin area [7]. The study sites represented the most typical soil catenas of the central part of Western Siberia [12].

**Table 2:** Characteristics of the study sites.

Study sites (m a.s.l.)	Area, km^2^	Location	Soils	Pits	Geological features of parent material	Climate [9]
1. The upland of Tobolsky Materik (75–77)	0.003	N 58°56′ E 69°10′	Retisols Gleyic	10	Sub-horizontally stratified well-sorted alluvial sands	Ever-humid taiga
2. The north of the Ishim Plain (123–126)	0.76	N 56°31′ E 67°32′	Anthric Phaeozems	8	Alluvial-lacustrine clay loams and clays with high contents of carbonates and residual salinity	Humid cold continental
3. The eastern part of the Trans-Urals region (135–142)	0.48	N 56°02′ E 63°35′	Anthric Chernozems	7	Alluvial-lacustrine clay loams and clays with high contents of carbonates and residual salinity	Humid cold continental

The soil pits were located in sequences from an interfluve down to the slope in order to analyse the redistribution of substances along the soil catenas. In total, there were 142 samples taken from 25 soil pits (Table 3). In soil samples, pHwater, the particle-size distribution, content of total organic carbon (TOC) and HMs were measured (Table 4). Data on HM content were compared to mean concentration within the upper part of the continental earth's crust or a Clark and in soils of the world (Table 5). A Clark of a chemical element and its mean concentration within the UCEC are synonyms.

**Table 3:** Raw data.

No	Pit no	Horizon	Sampling depth, cm	Particle size (mm), %						pH	TOC, %	Macroelements, %									Microelements, mg kg^−1^											
				<1	1–5	5–10	10–50	50–250	250–1000			Fe	Si	Al	Ca	Mg	Ti	Na	S	Р	Mn	Pb	Co	Ni	Sr	Cu	Zn	Cr	V	Rb	Ba	Th
Key area 1. Retisols																																
1	2	Folic	4–7	na	na	na	na	na	na	6.0	22.78	2.48	22	3.7	6.3	1.9	0.38	0.5	1.0	0.6	8967	80	21	125	271	44	596	191	64	65	1282	8
2	2	Folic	0–12	na	na	na	na	na	na	6.0	9.82	0.08	1	0.1	0.3	0.0	0.01	0.0	0.0	0.0	317	4	<10	3	10	3	18	5	2	2	37	<5
3	2	Folic	7–10	na	na	na	na	na	na	6.2	16.21	2.71	28	4.7	3.5	1.1	0.50	0.6	0.4	0.2	5848	61	23	54	225	30	346	143	87	72	1004	5
4	2	Albic	10–12	1.5	6.2	5.9	23	29	34	4.4	0.71	2.01	29	4.0	0.6	0.3	0.49	0.9	0.0	0.1	1541	11	11	14	128	13	52	89	88	58	466	<5
5	2	Umbric	19–29	3.2	13.3	10.6	56	17	0	4.7	1.55	2.98	33	5.6	0.6	0.5	0.66	0.6	0.0	0.0	565	17	6	21	159	45	53	179	119	65	519	<5
6	2	Umbric	30–35	3.5	15.5	11.4	55	15	0	4.8	0.86	3.82	31	6.5	0.7	0.8	0.66	0.6	0.0	0.0	426	17	15	36	165	61	62	170	136	70	524	7
7	2	Argic	55–60	3.9	13.4	8.2	56	19	0	4.9	0.44	3.96	31	6.6	0.7	0.8	0.65	0.6	<0.008	0.0	488	15	13	40	165	64	65	167	135	72	530	7
8	2	Argic	70–80	3.3	9.5	5.9	47	34	0	5.2	0.34	4.49	30	6.1	0.7	0.7	0.62	0.6	0.0	0.0	387	8	12	43	171	51	59	180	112	71	537	9
9	2	Gleyic	90–100	3.3	9.4	6.1	49	32	0.01	5.5	0.22	4.02	31	6.9	0.8	0.8	0.65	0.6	<0.008	0.0	682	14	25	42	165	65	68	185	134	70	544	7
10	3	Folic	0–5	na	na	na	na	na	na	5.9	20.16	3.95	24	4.1	3.9	1.6	0.42	0.5	0.6	0.5	10063	112	23	102	211	50	606	172	87	69	1136	<5
11	3	Umbric	5–9	1.3	5.3	4.7	19	32	38	5.2	3.55	5.54	33	5.7	1.0	0.7	0.61	0.6	0.1	0.2	4005	41	23	35	154	31	129	111	122	92	735	7
12	3	Albic	9–17	4.2	16.6	12.6	58	8	0.03	5.0	1.37	6.29	25	5.2	0.6	0.5	0.51	0.5	<0.008	0.1	1994	18	28	43	134	35	88	123	113	63	476	8
13	3	Umbric	19–21	4.5	21.3	14.8	51	9	0.02	5.0	2.04	5.28	28	5.7	0.7	0.6	0.56	0.6	0.0	0.1	1753	22	23	43	149	41	95	144	126	60	552	5
14	3	Argic	43–49	4.1	11.9	7.2	55	22	0	5.4	0.33	4.59	31	6.1	0.8	0.7	0.65	0.7	0.0	0.0	511	23	14	37	176	78	66	167	130	69	570	7
15	3	Gleyic	50–60	3.1	8.2	4.9	49	35	0	5.5	0.08	3.82	32	6.2	0.8	0.7	0.68	0.7	<0.008	0.0	426	18	11	32	179	68	58	167	124	70	550	6
16	3	Gleyic	90–100	3.0	7.9	3.9	42	43	0.03	5.7	0.05	3.88	32	6.8	0.8	0.8	0.69	0.7	<0.008	0.0	310	28	12	36	174	102	76	163	143	77	545	6
17	4	Folic	0–8	na	na	na	na	na	na	6.0	48.53	0.04	0	0.0	0.2	0.0	0.00	0.0	0.0	0.0	438	1	<10	3	6	2	37	3	1	1	77	<5
18	4	Folic	8–16	na	na	na	na	na	na	6.5	11.31	2.51	23	3.9	1.0	0.5	0.44	0.5	0.1	0.1	4610	22	17	25	125	20	109	97	96	55	762	7
19	4	Albic	21–31	3.5	15.6	11.2	47	22	0.02	5.3	1.14	3.79	32	6.0	0.8	0.7	0.68	0.7	0.0	0.1	635	27	13	28	168	63	58	168	131	74	636	<5
20	4	Umbric	34–44	4.0	14.7	12.5	60	9	0.01	5.8	2.04	4.04	28	5.7	0.8	0.7	0.59	0.6	0.0	0.1	708	11	21	37	154	24	62	137	112	64	545	<5
21	4	Gleyic	60–70	4.6	15.1	9.0	52	19	0	5.3	0.59	4.21	31	6.5	0.8	0.7	0.66	0.6	<0.008	0.0	643	11	15	43	169	64	65	166	123	73	549	7
22	4	Gleyic	90–100	5.6	14.4	8.5	48	24	0	5.8	0.50	8.38	27	5.9	0.8	0.7	0.59	0.4	<0.008	0.1	1635	25	26	68	179	36	68	156	127	79	539	11
23	4	Gleyic	120–130	4.8	11.1	6.1	46	33	0.05	6.1	0.43	3.93	31	6.7	0.9	0.8	0.68	0.6	<0.008	0.0	325	22	1	35	175	171	91	158	139	80	577	5
24	5	Folic	0–1	na	na	na	na	na	na	6.2	41.12	0.11	1	0.1	0.3	0.1	0.01	0.0	0.0	0.0	414	5	<10	4	13	3	29	7	3	2	58	<5
25	5	Umbric	1–2	na	na	na	na	na	na	5.4	6.33	2.17	25	4.3	0.5	0.4	0.48	0.6	0.0	0.1	454	16	11	17	115	25	85	107	104	50	447	<5
26	5	Albic	2–8	3.3	11.1	6.5	46	33	0	4.6	1.21	3.43	34	6.2	0.6	0.7	0.68	0.7	<0.008	0.1	364	11	13	20	158	30	47	169	127	67	559	<5
27	5	Umbric	8–18	3.5	10.1	6.9	54	26	0.02	5.0	1.18	3.13	30	6.3	0.7	0.7	0.61	0.6	<0.008	0.0	245	19	17	47	155	51	92	153	147	73	499	5
28	5	Gleyic	20–30	2.9	8.3	5.8	55	28	0	4.7	0.37	2.86	34	6.1	0.6	0.7	0.73	0.9	<0.008	0.0	310	17	9	20	170	43	50	159	132	71	534	6
29	5	Gleyic	45–50	2.4	6.8	3.6	42	45	0.01	4.8	0.38	3.03	33	6.5	0.7	0.7	0.70	0.7	0.0	0.0	263	23	14	26	174	65	58	193	118	74	527	8
30	5	Gleyic	80–90	3.3	10.0	5.8	36	45	0.02	5.2	0.35	3.72	31	7.0	0.8	0.8	0.67	0.6	<0.008	0.0	294	24	13	37	171	45	64	183	137	78	515	12
31	7	Folic	0–8	na	na	na	na	na	na	5.9	35.89	0.24	2	0.4	0.4	0.1	0.04	0.1	0.1	0.1	1094	10	<10	8	23	6	44	17	7	6	114	<5
32	7	Umbric	8–16	2.2	5.7	3.4	44	29	16.32	5.6	4.48	2.29	27	4.3	0.6	0.5	0.49	0.6	0.0	0.1	3958	21	17	18	123	25	79	106	94	63	613	<5
33	7	Albic	4–10	na	na	na	na	na	na	4.4	0.27	1.82	33	4.2	0.6	0.3	0.58	1.2	<0.008	0.0	570	7	7	11	138	13	43	119	107.07	73	487	6
34	7	Albic	10–20	3.7	12.9	6.5	42	35	0.05	4.8	0.64	3.11	34	6.2	0.6	0.7	0.71	0.8	<0.008	0.0	318	19	12	21	172	62	53	142	124	72	535	9
35	7	Gleyic	40–50	2.9	7.9	5.2	36	47	0.34	4.9	0.51	3.68	32	6.8	0.7	0.8	0.68	0.7	<0.008	0.0	325	24	15	38	170	42	62	190	142	75	525	8
36	7	Gleyic	90–100	2.8	9.7	7.3	49	32	0.01	5.0	0.51	3.82	32	6.8	0.7	0.8	0.67	0.7	<0.008	0.0	318	31	18	38	170	42	61	188	144	77	515	10
37	8	Folic	0–2	na	na	na	na	na	na	5.8	35.37	0.26	2	0.4	0.4	0.1	0.04	0.1	0.1	0.0	424	11	3	8	26	7	43	17	8	6	115	<5
38	8	Folic	2–12	na	na	na	na	na	na	5.6	20.55	1.06	10	1.9	0.6	0.2	0.18	0.2	0.0	0.1	881	17	8	11	69	15	79	46	43	26	308	<5
39	8	Albic	21–31	3.3	10.6	8.4	55	23	0	4.8	0.53	3.65	34	6.4	0.6	0.7	0.67	0.7	<0.008	0.0	364	20	17	20	164	35	54	149	131	69	509	5
40	8	Gleyic	45–55	2.8	6.7	3.9	40	47	0.02	5.0	0.35	3.51	33	6.8	0.7	0.8	0.66	0.7	<0.008	0.0	279	18	15	30	165	40	59	158	131	69	550	6
41	9	Folic	0–2	na	na	na	na	na	na	6.2	28.27	0.48	5	0.8	0.5	0.2	0.08	0.1	0.1	0.1	1209	14	0	10	34	6	50	30	17	11	164	<5
42	9	Folic	2–8	na	na	na	na	na	na	6.1	10.67	1.88	19	3.2	0.7	0.4	0.35	0.4	0.1	0.1	3761	28	17	23	98	20	92	90	63	48	461	<5
43	9	Albic	6–8	na	na	na	na	na	na	2.5	1.82	2.67	26	5.3	0.7	0.5	0.49	0.4	<0.008	0.2	518	28	10	20	124	19	63	102	112.86	78	489	4
44	9	Albic	8–12	na	na	na	na	na	na	4.2	0.98	2.38	24	4.2	0.4	0.3	0.45	0.6	<0.008	0.1	171	7	6	14	96	20	40	104	89.79	63	380	4
45	9	Albic	5–15	2.9	9.5	6.3	42	40	0	4.8	0.93	3.43	33	5.9	0.6	0.6	0.68	0.7	<0.008	0.1	1356	14	15	25	161	64	61	145	113	85	566	<5
46	9	Albic	12–16	na	na	na	na	na	na	4.2	0.53	2.50	31	4.3	0.4	0.3	0.50	0.9	0.0	0.1	211	12	5	13	119	19	33	107	100.61	63	424	5
47	9	Argic	27–37	3.0	7.2	4.8	52	33	0.01	4.9	0.42	3.44	33	6.0	0.7	0.7	0.71	0.7	<0.008	0.0	364	17	12	24	167	47	52	151	128	67	535	10
48	9	Gleyic	52–62	4.0	10.6	7.2	43	36	0	4.9	0.32	3.64	31	6.5	0.7	0.7	0.66	0.6	0.0	0.0	480	20	14	38	168	45	62	194	122	73	518	5
49	9	Gleyic	83–93	4.5	11.3	7.4	51	26	0.03	5.3	0.29	4.01	31	6.8	0.8	0.8	0.66	0.7	<0.008	0.0	434	24	16	39	172	38	69	175	130	72	539	10
50	10	Folic	0–4	na	na	na	na	na	na	6.0	21.42	0.71	8	1.3	0.6	0.3	0.14	0.2	0.1	0.1	2246	22	5	14	53	10	94	43	23	20	298	2
51	10	Folic	4–6	2.1	10.0	9.4	50	28	0	6.0	10.12	2.04	28	4.3	0.7	0.5	0.52	0.6	0.0	0.1	1751	15	13	16	135	22	66	119	90	72	564	4
52	10	Umbric	6–8	1.4	5.7	5.1	24	30	34	4.7	0.73	1.88	29	3.6	0.6	0.3	0.47	0.9	0.0	0.1	2158	11	14	15	115	11	47	104	85	61	470	<5
53	10	Albic	8–10	2.7	10.7	9.4	39	28	11	4.3	0.41	2.09	31	4.1	0.5	0.3	0.54	1.0	0.0	0.1	1488	10	12	15	130	13	40	117	112	69	481	5
54	10	Albic	10–12	4.0	14.1	13.5	41	28	0	4.8	0.59	2.72	34	6.0	0.6	0.7	0.70	0.9	<0.008	0.0	418	12	8	17	165	34	46	154	114	71	533	9
55	10	Argic	23–33	1.7	3.8	2.3	32	31	29.29	4.8	0.21	3.45	33	6.4	0.7	0.8	0.67	0.7	<0.008	0.0	349	15	12	31	174	67	61	150	134	73	538	9
56	10	Gleyic	50–60	3.3	8.3	5.3	38	45	0.03	4.8	0.24	3.89	32	6.4	0.7	0.8	0.69	0.7	<0.008	0.0	364	23	18	35	179	45	60	193	147	75	563	10
57	10	Gleyic	70–80	5.1	13.0	8.3	51	23	0.05	5.0	0.22	4.45	31	6.6	0.7	0.8	0.68	0.6	<0.008	0.0	341	11	12	42	177	52	69	187	146	77	569	<5
58	11	Folic	0–8	na	na	na	na	na	na	6.5	36.58	0.14	1	0.2	0.5	0.1	0.02	0.0	0.1	0.0	700	6	<10	8	21	6	46	12	4	3	96	<5
59	11	Folic	8–14	na	na	na	na	na	na	6.1	12.16	1.18	15	2.4	1.0	0.3	0.28	0.4	0.1	0.1	1997	20	9	15	95	13	85	70	50	36	427	3
60	11	Albic	11–14	na	na	na	na	na	na	4.5	0.47	1.80	32	4.1	0.6	0.3	0.56	1.1	0.0	0.1	1589	13	11	13	131	23	48	106	96.34	89	590	6
61	11	Umbric	14–18	3.0	11.2	9.0	50	26	0	5.0	4.51	2.23	32	5.0	0.6	0.5	0.61	0.8	0.0	0.0	777	20	14	14	151	16	73	126	110	66	518	6
62	11	Albic	34–44	2.5	7.4	6.3	51	33	0	4.8	0.67	2.79	34	6.0	0.7	0.7	0.71	0.8	<0.008	0.0	504	17	13	16	169	54	48	158	115	71	596	<5
63	11	Gleyic	60–70	3.2	8.1	5.2	39	45	0	4.9	0.53	3.51	32	6.5	0.7	0.8	0.67	0.7	<0.008	0.0	387	13	12	30	166	65	63	164	128	73	540	8
64	11	Gleyic	90–100	5.1	13.4	8.1	52	21	0.01	5.1	0.61	4.57	30	6.7	0.7	0.8	0.65	0.6	<0.008	0.0	387	25	10	42	174	75	75	174	161	76	529	5
65	12	Folic	0–4	na	na	na	na	na	na	5.8	28.54	0.45	5	0.8	0.6	0.2	0.08	0.1	0.1	0.1	913	18	<10	9	37	8	62	29	13	11	166	<5
66	12	Umbric	4–7	1.2	5.0	4.1	22	45	23	6.0	3.52	2.52	34	5.2	1.5	0.6	0.63	0.7	0.1	0.1	5576	30	26	22	187	14	86	118	109	88	952	7
67	12	Umbric	7–10	2.5	10.5	8.3	36	34	9	5.9	1.43	2.73	35	5.4	1.0	0.6	0.67	0.8	0.0	0.1	2657	27	21	15	171	13	44	121	130	88	669	5
68	12	Albic	17–27	2.9	7.7	4.2	35	51	0	4.9	0.70	3.27	33	6.5	0.7	0.7	0.70	0.7	<0.008	0.0	318	18	11	25	167	57	57	152	133	69	570	6
69	12	Argic	40–50	4.9	12.9	8.3	54	20	0.02	4.7	0.46	3.83	31	6.3	0.7	0.7	0.59	0.6	<0.008	0.0	606	20	17	34	154	37	88	169	130	65	547	5
70	12	Gleyic	80–90	3.8	9.4	5.7	41	40	0.09	5.7	0.46	4.69	31	6.5	0.8	0.8	0.64	0.6	<0.008	0.0	364	24	19	42	166	17	56	160	145	75	556	<5
Key area 2. Phaeozems																																
1	1	Mollic	7–17	7.2	31	15	43	4.2	0.0	6.9	2.0	4.4	33	7.6	1.2	1.2	0.79	0.67	0.03	0.08	1188	25	20	49	190	43	132	210	177	108	516	6
2	1	Mollic	23–32	7.8	30	15	43	4.7	0.0	7.0	5.5	4.5	33	7.6	1.2	1.2	0.77	0.59	0.06	0.08	1177	32	19	53	194	62	145	215	176	109	636	8
3	1	Argic	35–45	10.2	34	10	45	0.9	0.0	7.3	1.1	5.2	29	8.8	0.8	1.4	0.77	0.45	0.00	0.04	790	28	25	66	154	38	125	197	180	108	513	7
4	1	Argic	60–70	8.2	23	15	47	5.0	1.7	7.5	0.7	5.1	29	8.6	0.8	1.4	0.74	0.45	0.02	0.04	806	26	18	67	152	33	99	196	191	105	531	5
5	1	(Proto)calcic	100–105	10.8	27	14	45	2.7	0.1	8.0	0.3	4.5	28	7.7	3.4	1.6	0.71	0.45	0.03	0.04	782	28	24	60	194	45	86	161	162	91	537	6
6	1	Mollic	30–40	8.0	29	17	44	1.7	0.0	7.3	0.6	4.5	33	7.7	1.1	1.2	0.81	0.66	0.02	0.06	1190	24	18	58	184	35	130	183	187	109	568	12
7	2	Mollic	7–17	7.6	24	13	40	16.0	0.0	7.1	9.2	4.8	32	8.0	1.0	1.2	0.77	0.58	0.02	0.05	1112	36	20	59	178	65	157	289	208	113	551	7
8	2	Mollic	24–34	6.7	21	13	38	2.3	18.8	7.4	6.6	5.0	30	8.6	0.8	1.4	0.75	0.45	0.02	0.03	829	31	18	68	155	33	112	202	190	105	510	11
9	2	Argic	40–50	8.0	24	15	50	3.0	0.0	7.7	1.5	5.1	29	8.4	0.8	1.4	0.75	0.45	0.02	0.03	821	39	25	70	157	62	132	200	189	102	566	10
10	2	(Proto)calcic	60–70	10.7	28	8	37	1.4	14.5	8.1	0.6	4.1	27	7.4	5.4	1.6	0.68	0.45	0.05	0.03	775	26	18	59	215	43	73	154	135	85	463	<5
11	2	(Proto)calcic	110–120	5.4	12	6	16	0.9	59.5	8.0	0.2	4.0	27	6.8	6.0	1.5	0.65	0.45	0.04	0.03	705	27	19	57	243	35	70	154	121	81	421	12
12	3	Mollic	7–17	7.6	29	14	39	1.3	8.7	6.7	3.6	4.7	33	7.7	1.1	1.2	0.78	0.59	0.02	0.05	1202	22	26	57	187	37	135	212	182	112	605	11
13	3	Argic	30–40	8.1	29	19	42	1.6	0.0	6.8	0.2	5.7	28	8.8	0.8	1.4	0.71	0.37	0.03	0.03	821	28	18	76	153	35	107	214	189	114	513	8
14	6	Mollic	8–18	8.0	32	16	42	2.6	0.0	6.8	6.4	4.6	33	7.7	1.4	1.2	0.80	0.60	0.03	0.08	1229	24	17	63	195	44	142	232	176	111	602	7
15	6	Mollic	25–35	7.6	29	18	43	2.4	0.0	6.8	5.6	5.5	32	8.4	0.9	1.3	0.82	0.57	0.03	0.04	909	28	32	73	161	48	143	232	197	116	587	15
16	6	Argic	37–47	9.1	28	16	43	4.8	0.0	6.6	1.7	5.1	28	7.6	0.8	1.1	0.74	0.37	0.04	0.04	821	31	14	78	148	42	97	194	171	114	515	8
17	6	Argic	60–70	7.8	24	16	47	4.6	0.0	6.8	1.2	5.0	29	8.4	0.9	1.4	0.76	0.45	0.02	0.04	844	25	11	70	149	33	90	204	189	99	546	7
18	6	Argic	80–90	8.2	23	15	45	3.3	5.8	7.6	1.0	5.0	29	8.3	1.0	1.4	0.75	0.45	0.00	0.03	829	28	21	75	157	36	100	201	175	100	569	12
19	6	(Proto)calcic	95–105	7.7	20	11	31	2.7	27.2	7.9	0.9	4.6	28	7.7	2.6	1.4	0.73	0.45	0.03	0.03	837	27	19	69	175	45	110	187	156	97	530	8
20	7	Mollic	9–19	7.4	29	16	41	6.3	0.0	6.7	5.4	4.5	33	7.6	1.3	1.2	0.77	0.68	0.06	0.07	1202	32	22	54	186	60	155	222	152	105	505	9
21	7	Argic	35–45	9.6	29	18	42	1.3	0.0	7.6	0.9	5.0	29	8.3	1.4	1.4	0.76	0.37	0.02	0.04	806	33	22	67	159	38	94	183	185	105	498	12
22	7	(Proto)calcic	52–60	10.5	31	15	40	2.7	0.0	7.9	1.4	4.5	27	7.5	3.7	1.4	0.71	0.45	0.03	0.04	767	23	20	69	191	37	106	167	144	92	457	<5
23	7	Mollic	30–40	6.0	26	19	48	0.6	0.0	6.7	1.2	4.8	33	7.6	1.3	1.2	0.79	0.59	0.06	0.06	1128	26	23	68	186	43	122	209	141	114	534	<5
24	8	Mollic	0–10	9.2	33	17	40	1.2	0.0	6.7	4.9	4.8	33	7.8	1.1	1.2	0.77	0.59	0.03	0.05	1122	25	19	59	171	44	130	212	172	113	558	9
25	9	Mollic	8–18	7.8	31	16	43	2.0	0.0	7.2	6.0	4.5	33	7.7	1.3	1.1	0.76	0.68	0.02	0.07	1165	26	21	49	172	51	166	219	191	111	585	10
26	9	Mollic	25–35	9.3	33	15	42	0.8	0.0	7.2	5.2	4.6	33	7.7	1.3	1.1	0.78	0.59	0.02	0.06	1134	31	25	54	178	42	180	210	169	111	608	10
27	9	Argic	42–52	8.1	27	16	46	2.0	0.0	7.5	1.4	5.0	30	8.7	0.8	1.4	0.76	0.45	0.00	0.03	782	15	24	56	140	41	91	189	191	109	508	12
28	9	Argic	80–90	9.6	30	16	41	3.8	0.0	7.8	0.9	5.0	30	8.6	0.9	1.4	0.77	0.45	0.00	0.04	868	36	18	64	157	43	113	196	192	101	538	10
29	9	(Proto)calcic	90–100	12.2	32	13	41	1.3	0.0	8.0	1.0	4.2	27	7.4	5.2	1.4	0.70	0.45	0.04	0.04	728	17	18	59	201	34	74	153	136	82	483	9
30	11	Mollic	8–18	9.5	36	3	52	0.2	0.0	6.6	4.2	4.5	31	7.4	1.1	1.1	0.75	0.45	0.04	0.07	1085	34	21	61	159	45	104	212	171	104	539	5
31	11	Argic	30–40	8.6	30	17	43	0.4	0.0	6.6	1.1	5.3	29	8.5	0.8	1.3	0.74	0.37	0.02	0.04	798	29	18	67	137	32	99	188	182	104	516	7
32	11	Argic	60–70	9.2	30	9	51	1.2	0.0	6.5	1.2	5.0	30	8.6	0.9	1.4	0.76	0.45	0.02	0.04	837	24	25	65	144	40	142	206	188	100	566	9
33	11	Argic	95–105	12.0	32	13	42	0.5	0.0	7.5	0.9	5.0	32	8.2	1.1	1.3	0.78	0.73	0.00	0.03	902	32	20	70	163	41	163	192	173	102	536	10
34	11	(Proto)calcic	112–116	17.3	43	8	32	0.2	0.0	8.0	0.8	4.7	28	7.7	2.3	1.3	0.73	0.45	0.02	0.03	821	23	18	70	162	49	115	174	156	94	552	9
35	11	Mollic	25–35	8.4	32	18	40	1.6	0.0	6.5	3.9	4.9	32	8.4	1.1	1.2	0.81	0.67	0.00	0.06	932	30	18	58	160	61	182	207	186	109	593	8
36	12	Mollic	5–10	8.5	35	8	48	0.7	0.0	7.0	6.1	4.2	33	7.3	1.3	1.1	0.76	0.60	0.05	0.08	1243	28	24	47	173	62	158	241	181	105	624	9
37	12	Argic	30–40	9.6	33	17	39	0.7	0.0	6.8	1.4	5.2	28	8.1	0.8	1.2	0.76	0.37	0.02	0.04	829	34	17	71	144	87	119	204	184	112	541	10
38	12	Argic	60–70	8.7	27	17	45	1.7	0.0	6.8	1.1	5.0	30	8.5	0.9	1.4	0.77	0.45	0.02	0.04	906	27	28	65	144	37	85	203	189	99	529	<5
39	12	Argic	85–95	9.0	26	17	46	2.7	0.0	7.4	0.8	4.9	29	8.2	0.9	1.4	0.75	0.45	0.02	0.03	837	29	19	76	152	119	145	209	178	101	524	8
40	12	(Proto)calcic	100–105	15.8	30	10	44	0.0	0.0	7.6	0.8	4.7	30	8.3	1.2	1.5	0.76	0.52	0.02	0.03	868	21	29	57	164	38	87	169	190	97	560	<5
41	12	Mollic	25–35	8.4	27	12	51	2.1	0.0	6.3	3.8	4.9	32	8.2	1.1	1.2	0.81	0.67	0.00	0.05	1053	32	22	62	168	45	130	195	200	112	554	15
42	14	Mollic	0–10	9.3	38	16	36	0.8	0.0	7.4	2.3	4.5	33	7.6	1.5	1.2	0.76	0.68	0.05	0.08	1143	28	22	55	184	51	158	279	179	105	596	8
43	15	Mollic	0–10	8.5	35	15	39	2.8	0.0	6.8	4.6	4.3	34	7.1	1.1	1.1	0.78	0.58	0.03	0.05	1228	22	29	47	172	60	135	226	156	107	526	11
44	16	Mollic	10–20	5.7	24	11	57	2.6	0.0	6.1	6.2	3.6	35	6.2	1.1	0.8	0.77	0.68	0.03	0.08	1400	17	17	39	158	58	127	153	152	96	588	<5
45	16	Mollic	20–30	6.1	26	8	51	2.3	5.9	6.2	4.9	3.9	35	6.6	1.0	0.9	0.82	0.66	0.09	0.06	1358	33	20	46	160	58	107	173	186	99	583	10
46	16	Argic	30–40	8.0	30	15	45	3.0	0.0	6.2	1.8	5.3	29	8.3	0.8	1.2	0.74	0.37	0.02	0.03	899	31	25	70	131	47	125	213	185	108	504	9
47	16	Argic	50–60	7.8	26	16	46	0.9	3.1	6.2	1.0	5.2	29	8.6	0.8	1.3	0.74	0.37	0.02	0.03	883	35	25	70	134	44	115	189	195	107	541	13
48	16	Argic	80–90	8.4	28	13	43	7.0	0.1	6.7	0.8	5.0	29	8.3	0.9	1.3	0.76	0.45	0.02	0.03	782	30	19	71	139	39	86	205	174	101	526	9
49	16	(Proto)calcic	95–105	13.4	36	11	39	0.9	0.0	8.0	0.8	4.5	27	7.2	3.9	1.3	0.71	0.37	0.03	0.03	751	37	24	71	167	43	78	153	152	92	472	11
50	17	Mollic	10–20	9.1	34	16	38	2.5	0.0	6.7	4.9	4.6	33	7.6	1.2	1.1	0.77	0.67	0.02	0.05	1170	27	17	53	173	53	117	248	184	110	595	15
51	17	Argic	41–51	9.1	29	13	46	3.1	0.0	6.8	0.9	4.9	29	8.2	0.9	1.3	0.76	0.45	0.02	0.03	775	18	26	69	154	37	82	212	180	97	534	9
Key area 3. Chernozems																																
1	1	Chernic	5–10	6.4	27	17	39	11	0.01	6.5	7.4	3.4	24	5.6	1.0	0.8	0.50	0.44	0.03	0.06	724	16	20	40	139	24	117	132	127	409	77	5.1
2	1	Chernic	21–31	6.4	27	16	35	15	0.21	6.6	7.3	3.5	24	5.7	1.0	0.8	0.52	0.45	0.03	0.05	720	18	16	38	144	28	132	141	130	404	77	10.3
3	1	Mollic-argic	38–48	7.1	27	18	37	11	0.04	7.7	3.3	4.2	27	6.6	0.8	1.0	0.58	0.47	0.03	0.04	669	23	18	51	146	24	114	166	157	459	82	4.5
4	1	(Proto)calcic	80–90	10.2	27	14	33	16	0.19	8.3	1.5	4.3	25	6.8	5.0	1.2	0.63	0.30	0.07	0.03	651	27	13	70	183	33	66	178	119	410	79	9.0
5	2	Chernic	5–10	7.2	29	15	38	10	0.06	6.6	6.8	3.6	24	5.8	1.0	0.9	0.52	0.51	0.02	0.06	756	18	17	45	149	33	138	158	124	413	74	8.6
6	2	Chernic	27–37	8.0	31	19	39	3	0.02	6.7	5.9	3.8	25	6.1	1.0	1.0	0.54	0.45	0.03	0.06	766	13	11	52	150	30	115	185	143	437	79	4.4
7	2	Mollic-argic	43–53	16.1	40	12	29	3	0	7.5	2.6	5.0	27	7.2	1.0	1.3	0.69	0.37	0.08	0.03	844	35	14	88	172	43	88	217	171	489	93	12.0
8	2	(Proto)calcic	65–75	7.3	24	15	38	16	0.44	8.3	1.3	4.3	26	6.8	6.0	1.4	0.62	0.45	0.06	0.04	759	27	19	70	222	44	71	166	139	407	79	7.0
9	5	Chernic	6–16	7.1	31	16	44	2	0.03	6.7	10.2	3.4	24	5.4	1.1	0.8	0.50	0.44	0.04	0.07	782	17	18	42	147	31	100	151	121	429	75	6.8
10	5	Chernic	36–36	6.6	28	18	42	5	0.05	7.6	5.0	4.0	25	6.4	1.0	1.0	0.57	0.46	0.05	0.05	769	31	19	50	149	28	115	165	149	451	82	10.6
11	5	Mollic-argic	42–52	7.1	26	17	39	10	0.11	8.1	3.7	4.1	27	6.5	1.0	1.1	0.57	0.47	0.06	0.04	745	25	15	54	148	31	116	187	161	443	80	9.1
12	5	(Proto)calcic	75–85	10.2	28	14	35	13	0.04	8.5	0.5	4.2	25	6.8	5.4	1.3	0.63	0.37	0.08	0.04	713	15	19	67	188	30	68	186	120	400	80	7.0
13	6	Chernic	8–18	7.0	28	17	41	6	0.09	6.7	8.5	3.6	24	5.5	1.1	0.9	0.50	0.38	0.06	0.07	819	25	13	48	153	30	89	156	130	388	76	4.3
14	6	Chernic	35–45	8.1	28	17	41	6	0.08	7.6	2.9	4.2	26	6.7	0.9	1.2	0.59	0.47	0.06	0.04	800	33	23	57	153	26	96	184	150	435	80	10.9
15	6	(Proto)calcic	75–85	12.4	34	15	36	3	0.02	8.4	0.9	4.3	25	6.8	5.8	1.5	0.63	0.37	0.07	0.03	720	18	17	68	219	35	69	165	133	358	81	6.0
16	7	Chernic	4–14	7.4	29	17	39	8	0.05	6.6	6.7	3.6	25	5.8	1.0	0.9	0.52	0.45	0.03	0.06	835	23	13	47	151	31	93	139	144	404	77	0.0
17	7	Chernic	19–29	5.3	21	13	28	6	26.64	6.9	6.3	3.7	25	5.9	1.0	1.0	0.54	0.45	0.02	0.06	809	24	13	48	143	23	110	174	124	441	77	12.2
18	7	Mollic-argic	33–43	11.3	34	16	36	2	0.05	7.7	1.1	4.9	28	7.7	1.2	1.4	0.69	0.37	0.06	0.03	852	23	22	84	170	33	83	214	177	507	93	10.0
19	7	(Proto)calcic	75–85	35.4	40	9	12	3	0.08	8.5	0.4	4.3	25	6.8	5.3	1.4	0.62	0.37	0.07	0.03	775	21	13	69	214	38	71	170	124	417	80	<5
20	8	Chernic	5–15	7.7	32	7	46	7	0.3	6.4	8.5	3.3	23	5.3	1.1	0.8	0.47	0.44	0.03	0.06	748	19	13	41	149	31	118	150	129	416	76	5.0
21	8	Chernic	20–30	4.6	19	9	31	5	29.98	6.7	7.1	3.4	23	5.3	1.1	0.8	0.49	0.44	0.03	0.06	729	23	14	39	147	30	119	168	126	366	79	0.0
22	8	Mollic-argic	45–55	9.9	26	10	43	11	0.38	7.0	2.0	4.2	27	6.5	0.9	1.0	0.60	0.54	0.02	0.03	743	22	24	54	153	30	113	209	159	466	78	9.1
23	8	Mollic-argic	60–65	9.3	30	16	39	5	0.02	7.6	0.8	4.1	28	6.4	0.9	1.1	0.58	0.54	0.03	0.03	724	27	23	49	151	28	147	190	158	476	76	7.3
24	8	(Proto)calcic	87–97	14.6	41	14	30	0	0.02	8.3	0.9	4.3	25	6.8	5.3	1.4	0.64	0.37	0.06	0.03	713	25	18	68	210	39	74	169	133	387	82	<5
25	9	Chernic	5–15	7.2	31	11	42	8	0.24	6.8	6.9	3.5	23	5.5	1.1	0.9	0.50	0.44	0.04	0.07	767	19	18	43	149	34	129	155	136	392	75	6.8
26	9	Chernic	25–35	6.8	30	11	42	10	0.24	6.9	7.4	3.6	23	5.6	1.2	0.9	0.50	0.44	0.04	0.06	752	19	15	46	151	38	129	157	140	397	78	6.0
27	9	(Proto)calcic	40–50	9.1	29	11	40	10	0.15	7.4	2.5	4.9	27	6.9	1.0	1.2	0.67	0.37	0.08	0.03	798	33	18	84	165	80	95	181	168	494	90	9.0
28	9	(Proto)calcic	62–72	21.6	31	8	35	4	0.3	8.3	0.8	4.4	24	6.5	5.2	1.3	0.62	0.37	0.08	0.04	689	24	20	75	212	32	72	181	133	380	79	9.0

na – not avalaible. Pit location is presented in Fig. 1.

**Table 4:** Root mean square deviation (relative %) for X-Ray fluorescence analysis.

Concentration, %	Na_2_O	MgO	Al_2_O_3_	SiO_2_	K_2_O	CaO	TiO_2_	MnO	Fe_2_O_3_	P_2_O_5_	S	V	Cr	Co,Ni,Cu, Zn	Rb, Sr	Ba	Pb	Th, U
60–100	na	ОС.	0.6	**0.4**	na	na	na	na	0.4	na	na	na	na	na	na	na	na	na
50–60	na	0.7	0.6	**0.4**	na	0.6	0.4	0.4	0.4	na	na	na	na	na	na	na	na	na
40–50	na	0.9	0.8	**0.5**	na	0.7	0.5	0.5	0.5	na	0.4	na	0.5	na	na	na	na	na
30–40	na	0.9	1.1	0.7	na	0.9	0.6	0.6	0.6	0.6	0.5	na	0.6	na	na	na	na	na
20–30	na	1.3	1.4	1.0	na	1.1	0.8	0.7	0.7	0.8	0.6	na	0.8	0.7	0.6	na	na	na
10–20	1.8	1.7	**1.8**	1.6	1.8	1.6	1.1	1.1	1.1	1.4	0.8	na	1.0	1.1	0.9	na	1.1	na
5–10	2.7	2.3	**2.7**	2.5	2.7	**2.5**	1.8	2.2	**2.2**	1.6	1.7	na	1.3	1.4	1.1	1.4	1.4	na
2–5	4.0	**3.3**	4.0	3.4	**4.0**	**3.4**	2.7	3.5	**3.5**	2.5	2.7	3.0	1.8	2.3	1.6	2.4	2.4	na
1–2	5.0	**4.5**	5.5	4.7	**5.0**	**4.7**	**3.5**	5.0	**5.0**	2.2	3.8	4.0	2.3	3.4	2.5	3.4	3.4	2.0
0.5–1	**6.0**	**6.5**	7.5	6.0	6.0	**6.0**	**4.5**	6.5	6.5	3.0	5.0	5.0	3.0	4.5	3.5	4.5	4.5	2.7
0.2–0.5	**8.0**	8.0	10.0	8.5	8.0	8.5	5.5	**8.5**	8.5	**4.1**	6.0	6.0	3.5	5.5	4.5	5.5	5.5	4.0
0.1–0.2	10.0	10.5	12.5	10.5	10.0	10.5	7.0	**10.5**	10.5	**4.7**	7.0	8.0	4.3	7.0	6.0	**7.0**	7.0	5.0
(1000–500) × 10^−4^	12.0	13.5	14.0	13.5	12.0	13.5	9.0	**12.5**	12.5	**6.0**	**8.5**	9.0	5.0	9.0	8.0	**9.0**	8.5	6.5
(500–200) × 10^−4^	14.0	15.0	15.0	15.0	14.0	15.0	10.5	14.0	14.0	8.0	**10.5**	**10.5**	**5.5**	10.5	**9.0**	**10.5**	10.5	9.0
(200–100) × 10^−4^	15.0	15.0	15.0	15.0	15.0	15.0	13.5	15.0	15.0	10.5	**13.0**	**12.5**	**7.0**	**12.5**	**10.5**	12.5	12.5	12.5
(100–50) × 10^−4^	na	na	na	na	na	15.0	14.5	15.0	15.0	12.0	na	**15.0**	9.0	**13.5**	**12.0**	15.0	15.0	15.0
(50–20) × 10^−4^	na	na	na	na	na	na	15.0	15.0	na	12.5	na	15.0	10.5	**15.0**	13.5	na	**15.0**	15.0
(20–10) × 10^−4^	na	na	na	na	na	na	na	15.0	na	na	na	na	na	**20.0**	15.0	na	**15.0**	**15.0**

na – not avalaible. Root mean square deviations corresponding to concentration in samples analysed are in bold.

**Table 5:** Descriptive statistics results for grain size fractions (G1 – G6), pH, TOC, macroelements (%) and heavy metals (mg kg^−1^).

Parameter	Min	P_10_	P_25_	M	GM	P_50_	P_75_	P_90_	Max	V	SD	Cv,%	Skewness	Kurtosis	N
Key site 1. Retisols															
Albic															
G1	1,5	2,5	2,9	33,7	9,1	3,6	100	100	100	2195	46,9	139	0,89	−1,39	16
G2	6,2	7,4	10,1	39,2	21,5	13,5	100	100	100	1859	43,1	110	0,88	−1,39	16
G3	4,2	5,9	6,4	37,2	17,3	10,3	100	100	100	1978	44,5	120	0,88	−1,39	16
G4	23,3	34,7	41,3	61,5	55,6	49,4	100	100	100	820	28,6	47	0,61	−1,38	16
G5	8,5	22,3	27,7	52,1	41,4	34,0	100	100	100	1235	35,2	67	0,68	−1,42	16
G6	0	0	0	34,4		0,04	100	100	100	2225	47,2	137	0,8	−1,47	16
pH	2,5	4,2	4,35	4,51	4,46	4,69	4,82	5	5,25	0,38	0,61	14	−2,48	8,11	16
TOC	0,27	0,41	0,53	0,81	0,72	0,69	1,06	1,37	1,82	0,17	0,41	50	1,11	1,06	16
Fe	1,8	1,82	2,23	2,98	2,83	2,75	3,43	3,79	6,29	1,19	1,09	36	1,88	5,17	16
Si	23,9	24,5	29,6	31,1	30,9	32,5	33,6	34,2	34,4	12,0	3,5	11	−1,22	0,2	16
Al	3,98	4,07	4,16	5,29	5,2	5,59	6,14	6,41	6,46	0,97	0,99	19	−0,26	−1,87	16
Ca	0,4	0,43	0,57	0,59	0,59	0,59	0,65	0,7	0,79	0,01	0,09	16	−0,28	1,04	16
Mg	0,27	0,29	0,32	0,52	0,48	0,56	0,66	0,72	0,72	0,03	0,18	35	−0,24	−1,86	16
Ti	0,45	0,49	0,51	0,6	0,6	0,62	0,69	0,71	0,71	0,01	0,1	16	−0,23	−1,75	16
Na	0,45	0,45	0,67	0,79	0,76	0,78	0,9	1,07	1,17	0,04	0,2	26	0,08	−0,19	16
S	0,02	0,02	0,02	69,44	6,95	101	101	101	101	2337	48	70	−0,9	−1,39	16
Р	0,03	0,03	0,04	0,06	0,06	0,05	0,07	0,12	0,19	0	0,04	64	2,26	5,92	16
Mn	171	211	341	772	585	511	1422	1589	1994	356583	597	77	0,92	−0,73	16
Pb	6,5	7,5	10,8	15,3	14,1	13,7	18,7	27	28,2	39,7	6,3	41	0,74	0,04	16
Co	5,4	5,7	8,8	11,9	11,0	11,4	13	17	28,0	28,9	5,4	45	1,78	4,98	16
Ni	11,2	12,7	14,0	19,6	18,4	18,5	23	28	42,9	63,1	7,9	40	1,77	4,09	16
Sr	96	119	129	145	143	148	166	169	172	527	23	16	−0,53	−0,72	16
Cu	13	13	19	35	30	32	56	63	64	371	19	56	0,47	−1,4	16
Zn	33	40	44	52	51	50	58	63	88	162	13	25	1,46	3,81	16
Cr	89	102	107	132	129	133	153	168	169	672	26	20	−0,06	−1,42	16
V	88	90	104	113	112	113	126	131	133	201	14	13	−0,3	−0,7	16
Rb	58	63	65	71	70	70	73	85	89	65	8	11	0,82	0,85	16
Ba	380	424	479	519	514	521	568	596	636	4522	67	13	−0,27	−0,17	16
Th	3,7	4,1	5,2	36	14	6,9	101	101	101	2071	46	128	0,89	−1,39	16
Argic															
G1	1,7	1,7	3,0	3,5	3,3	3,6	4,1	4,9	4,9	1,2	1,1	32	−0,54	0,61	6
G2	3,8	3,8	7,2	9,8	9,0	10,7	12,9	13,4	13,4	13,9	3,7	38	−0,83	−0,42	6
G3	2,3	2,3	4,8	6,1	5,6	6,6	8,2	8,3	8,3	5,2	2,3	37	−0,91	0,08	6
G4	31,7	31,7	47,3	49,4	48,5	53,1	55,1	56,0	56,0	84,6	9,2	19	−1,9	3,64	6
G5	18,6	18,6	19,9	26,4	25,6	26,5	33,0	34,1	34,1	49,9	7,1	27	−0,02	−2,92	6
G6	0	0	0	4,89		0,01	0,02	29	29	143	12	245	2,45	6	6
pH	4,7	4,7	4,8	5,0	5,0	4,9	5,2	5,4	5,4	0,1	0,3	5	0,78	−1,08	6
TOC	0,21	0,21	0,33	0,37	0,36	0,38	0,44	0,46	0,46	0,01	0,09	25	−1,03	0,77	6
Fe	3,4	3,4	3,5	4,0	3,9	3,9	4,5	4,6	4,6	0,2	0,5	12	0,31	−1,85	6
Si	30,1	30,1	30,5	31,3	31,3	30,9	32,6	33,0	33,0	1,4	1,2	4	0,73	−1,58	6
Al	6,0	6,0	6,1	6,2	6,2	6,2	6,4	6,6	6,6	0,04	0,2	3	0,55	−0,24	6
Ca	0,65	0,65	0,67	0,7	0,7	0,69	0,73	0,76	0,76	0	0,04	6	0,72	−0,26	6
Mg	0,66	0,66	0,66	0,72	0,72	0,73	0,78	0,78	0,78	0	0,05	7	−0,2	−1,94	6
Ti	0,59	0,59	0,62	0,65	0,65	0,65	0,67	0,71	0,71	0	0,04	6	0,15	0,06	6
Na	0,59	0,59	0,59	0,66	0,66	0,65	0,74	0,74	0,74	0	0,07	10	0,37	−2,12	6
S	0,02	0,02	0,02	67,34	5,89	101	101	101	101	2719	52	77	−0,97	−1,88	6
Р	0,03	0,03	0,03	0,03	0,03	0,03	0,04	0,04	0,04	0	0,01	19	0,67	−1,93	6
Mn	349	349	364	451	442	438	511	606	606	10228	101	22	0,59	−1,05	6
Pb	8	8	15	16	16	16	19,79	23	23	25,97	5,1	31	−0,53	0,93	6
Co	12	12	12	13	13	13	14	17	17	3,8	2,0	15	1,67	2,63	6
Ni	24	24	31	35	34	35	40	43	43	46	6,8	20	−0,6	0,03	6
Sr	154	154	165	168	168	169	174	176	176	65	8	5	−1,15	1,4	6
Cu	37	37	47	57	56	58	67	78	78	227	15	26	0,01	−1,02	6
Zn	52	52	59	65	64	63	66	88	88	147	12	19	1,5	3,13	6
Cr	150	150	151	164	164	167	169	180	180	132	12	7	−0,12	−0,93	6
V	112	112	128	128	128	130	134	135	135	70	8,4	7	−1,91	4,07	6
Rb	65	65	67	70	69	70	72	73	73	9	3,1	4	−0,45	−1,29	6
Ba	530	530	535	543	543	538	547	570	570	207	14	3	1,74	3,21	6
Th	4,7	4,7	7	7,8	7,6	8	9	10	10	4	1,9	25	−0,65	−0,22	6
Fiolic															
pH	5,6	5,8	5,9	6,0	6,0	6,0	6,2	6,5	6,5	0,1	0,2	4	0,53	0,36	17
TOC	9,8	10,1	12,2	24,1	21,2	21,4	35,4	41,1	48,5	145,7	12,1	50	0,5	−0,8	17
Fe	0,04	0,1	0,2	1,2	0,6	0,7	2,0	2,7	4,0	1,4	1,2	99	0,93	−0,07	17
Si	0,3	0,8	2,2	11,4	5,6	8,0	22,3	27,5	27,6	108,2	10,4	91	0,44	−1,56	17
Al	0,04	0,1	0,4	1,9	0,9	1,3	3,7	4,3	4,7	3,0	1,7	91	0,42	−1,6	17
Ca	0,2	0,3	0,4	1,3	0,7	0,6	1,0	3,9	6,3	2,8	1,7	132	2,24	4,5	17
Mg	0,03	0,05	0,11	0,44	0,24	0,23	0,47	1,57	1,85	0,29	0,54	122	1,86	2,56	17
Ti	0	0,01	0,04	0,2	0,1	0,14	0,38	0,5	0,52	0,04	0,19	93	0,46	−1,51	17
Na	0,01	0,01	0,06	0,26	0,13	0,18	0,48	0,6	0,65	0,05	0,23	89	0,4	−1,53	17
S	0,03	0,03	0,05	0,17	0,08	0,06	0,09	0,64	0,96	0,07	0,26	156	2,42	5,34	17
Р	0,01	0,03	0,04	0,12	0,07	0,06	0,09	0,47	0,57	0,03	0,16	129	2,24	4,13	17
Mn	317	414	700	2684	1525	1209	3761	8967	10063	9141989	3024	113	1,57	1,57	17
Pb	1,07	3,7	10	26	15	18	22	80	112	899	30	115	2,01	3,62	17
Co	0	2,9	9,1	44		21	101	101	101	1932	44	100	0,6	−1,75	17
Ni	2,65	3	7,7	26	14	11	23	102	125	1260	35	138	2,2	4,04	17
Sr	5,99	10	23	85	51	53	125	225	271	6816	83	97	1,13	0,23	17
Cu	2,25	2,7	6,0	16	11	10	20	44	50	198	14	91	1,41	1,31	17
Zn	18,25	29	44	141	81	66	94	596	606	35296	188	133	2,07	3,08	17
Cr	2,57	5,1	17	64	35	43	97	172	191	3730	61	95	0,91	−0,39	17
V	1,07	2,2	7,3	39	19	23	64	90	96	1289	36	92	0,46	−1,5	17
Rb	0,9	1,8	5,9	30	15	20	55	72	72	770	28	93	0,5	−1,51	17
Ba	37	58	114	416	252	298	564	1136	1282	161256	402	97	1,1	0,02	17
Th	na	na	na	na	na	na	na	na	na	na	na	na	na	na	na
Gleyic															
G1	2,4	2,8	2,9	3,7	3,6	3,3	4,6	5,1	5,6	0,9	0,9	26	0,63	−0,98	19
G2	6,7	6,8	8,1	10,0	9,7	9,4	11,3	14,4	15,1	6,2	2,5	25	0,74	−0,43	19
G3	3,6	3,9	5,2	6,2	6,0	5,8	7,4	8,5	9,0	2,6	1,6	26	0,17	−0,93	19
G4	35,9	36,2	39,6	45,1	44,8	45,5	51,0	52,4	54,8	36,4	6,0	13	−0,07	−1,4	19
G5	18,9	21,2	25,7	35,0	33,6	34,9	45,0	46,9	47,5	94,3	9,7	28	−0,2	−1,42	19
G6	0	0	0	0		0	0	0,1	0,3	0	0,1	209	3,75	15,09	19
pH	4,7	4,8	4,9	5,2	5,2	5,1	5,5	5,8	6,1	0,2	0,4	8	0,72	−0,39	19
TOC	0,05	0,08	0,24	0,37	0,32	0,37	0,51	0,59	0,61	0,03	0,16	43	−0,42	−0,42	19
Fe	2,9	3,0	3,6	4,1	4,0	3,9	4,2	4,7	8,4	1,3	1,1	28	3,22	12,51	19
Si	27	30	31	31	31	31	32	33	34	1,9	1,4	4	−1,54	5,79	19
Al	5,9	6,1	6,5	6,6	6,6	6,6	6,8	6,9	7,0	0,1	0,3	4	−0,79	0,23	19
Ca	0,63	0,65	0,71	0,75	0,75	0,74	0,78	0,83	0,87	0	0,06	8	−0,07	0,29	19
Mg	0,66	0,66	0,72	0,78	0,77	0,78	0,84	0,84	0,84	0	0,06	8	−0,58	−0,73	19
Ti	0,59	0,64	0,66	0,67	0,67	0,67	0,68	0,7	0,73	0	0,03	4	−0,77	2,9	19
Na	0,45	0,59	0,59	0,66	0,65	0,67	0,74	0,74	0,89	0,01	0,1	15	0,32	1,22	19
S	0,02	0,02	101	90	41	101	101	101	101	1014	32	35	−2,8	6,51	19
Р	0,02	0,02	0,03	0,03	0,03	0,03	0,03	0,04	0,08	0	0,01	36	2,87	10,37	19
Mn	263	279	310	451	402	364	434	682	1635	94803	308	68	3,5	13,45	19
Pb	11	11	17	21	20	23	24	28	31	32	5,6	27	−0,35	−0,57	19
Co	1	9	12	14	13	14	18	25	26	31	5,6	38	0,03	1,71	19
Ni	20	26	32	38	36	38	42	43	68	91	10	25	1,49	5,62	19
Sr	165	165	168	172	172	171	175	179	179	22	4,7	3	0,17	−1,07	19
Cu	17	36	42	59	53	45	65	102	171	1079	33	56	2,4	7,43	19
Zn	50	56	59	65	64	63	69	76	91	81	9,0	14	1,29	2,99	19
Cr	156	158	160	174	174	174	188	193	194	196	14	8	0,13	−1,72	19
V	118	122	127	135	135	134	144	147	161	119	11	8	0,45	0,01	19
Rb	69	70	72	74	74	75	77	79	80	10	3,2	4	−0,05	−0,86	19
Ba	515	515	527	541	541	540	550	569	577	310	18	3	0,27	−0,42	19
Th	5	5	6	17	10	8	10	101	101	871	30	169	2,77	6,41	19
Umbric															
G1	1,2	1,3	1,8	11,0	3,4	3,1	3,8	4,5	100	805	28,4	259	3,46	11,96	12
G2	5,1	5,3	5,7	18,3	11,7	10,8	15,1	21,3	100	703	26,5	145	3,25	10,93	12
G3	3,4	4,1	4,9	16,0	9,2	8,7	12,0	14,8	100	730	27,0	169	3,36	11,45	12
G4	19,0	21,8	30,1	47,6	43,0	50,6	55,2	59,5	100	485	22,0	46	1,03	2,39	12
G5	8,6	9,3	16,1	31,0	25,0	27,5	33,1	45,1	100	599	24,5	79	2,36	6,78	12
G6	0,0	0,0	0,0	18,4		4,4	28,3	37,7	100	871	29,5	161	2,28	5,84	12
pH	4,7	4,7	4,9	5,3	5,2	5,1	5,7	5,9	6,0	0,2	0,5	9	0,39	−1,43	12
TOC	0,7	0,9	1,3	2,7	2,2	2,0	4,0	4,5	6,3	3,1	1,8	66	0,8	−0,25	12
Fe	1,88	2,17	2,26	3,22	3,03	2,85	3,93	5,28	5,54	1,48	1,21	38	1	−0,09	12
Si	25,26	27,03	28,19	30,46	30,32	30,58	32,94	33,84	35,23	9,1	3,02	10	−0,11	−0,87	12
Al	3,59	4,26	4,63	5,27	5,2	5,5	5,73	6,3	6,49	0,74	0,86	16	−0,62	−0,2	12
Ca	0,55	0,58	0,58	0,77	0,73	0,66	0,89	0,99	1,52	0,08	0,28	36	2,06	4,64	12
Mg	0,3	0,4	0,48	0,57	0,56	0,59	0,69	0,75	0,75	0,02	0,14	25	−0,54	−0,26	12
Ti	0,47	0,48	0,52	0,59	0,58	0,61	0,64	0,66	0,67	0	0,07	12	−0,62	−1,01	12
Na	0,56	0,58	0,59	0,68	0,67	0,62	0,79	0,84	0,89	0,01	0,12	17	0,94	−0,78	12
S	0,02	0,02	0,02	8,45	0,06	0,03	0,06	0,11	101	849	29	345	3,46	12	12
Р	0,02	0,03	0,04	0,07	0,06	0,07	0,08	0,09	0,22	0	0,05	74	2,43	7,22	12
Mn	245	426	510	1940	1239	1265	3307	4005	5576	3116490	1765	91	0,95	−0,21	12
Pb	11	11	16	21	20	20	24	30	41	68	8,3	39	1,23	1,87	12
Co	6,0	11	14	17	16	17	22	23	26	33	5,8	33	−0,36	−0,26	12
Ni	14	15	16	27	24	22	36	43	47	146	12	45	0,54	−1,35	12
Sr	115	115	136	150	148	154	162	171	187	484	22	15	−0,38	−0,28	12
Cu	11	13	15	30	26	25	43	51	61	269	16	55	0,67	−0,68	12
Zn	44	47	58	76	72	76	89	95	129	580	24	32	0,76	0,84	12
Cr	104	106	109	131	129	124	149	170	179	650	26	19	0,75	−0,61	12
V	85	94	106	116	115	115	128	136	147	308	18	15	−0,03	−0,24	12
Rb	50	60	62	70	69	65	80	88	92	164	13	18	0,61	−0,52	12
Ba	447	470	509	587	574	534	641	735	952	20013	141	24	1,79	3,46	12
Th	4,6	4,7	5,3	45	19	6,9	101	101	101	2406	49	108	0,39	−2,26	12
Key site 2. Phaeozems															
(Proto)calcic															
G1	5,4	5,4	10,5	11,5	11,0	10,8	13,4	17,3	17,3	14	3,71	32	−0,05	−0,19	9
G2	12,5	12,5	26,6	28,9	27,5	30,2	32,3	42,6	42,6	76	8,75	30	−0,52	0,8	9
G3	6,0	6,0	8,3	10,7	10,3	10,5	13,3	15,0	15,0	9,6	3,11	29	−0,01	−1,09	9
G4	15,8	15,8	32,3	36,2	34,8	39,2	40,9	45,3	45,3	80	8,94	25	−1,62	3,12	9
G5	0	0	0,9	1,4		1,3	2,7	2,7	2,7	1,2	1,07	75	0,17	−1,53	9
G6	0	0	0	11,3		0	14,5	59,5	59,5	419	20,47	182	2,02	3,96	9
pH	7,6	7,6	7,9	7,9	7,9	8,0	8,0	8,1	8,1	0,02	0,14	2	−1,91	4,32	9
TOC	0,25	0,25	0,56	0,76	0,67	0,78	0,93	1,37	1,37	0,12	0,35	46	0,16	0,01	9
Fe	3,98	3,98	4,24	4,43	4,42	4,5	4,63	4,69	4,69	0,07	0,26	6	−0,82	−0,73	9
Si	27	27	27	28	28	27	28	30	30	1,3	1,2	4	1,15	1,34	9
Al	6,8	6,8	7,4	7,5	7,5	7,5	7,7	8,3	8,3	0,18	0,42	6	0,14	1,3	9
Ca	1,2	1,2	2,6	3,7	3,4	3,7	5,2	6,0	6,0	2,4	1,6	42	−0,08	−0,87	9
Mg	1,3	1,3	1,4	1,4	1,4	1,4	1,5	1,6	1,6	0,01	0,11	8	−0,58	−0,84	9
Ti	0,65	0,65	0,7	0,71	0,71	0,71	0,73	0,76	0,76	0	0,03	4	−0,23	0,49	9
Na	0,37	0,37	0,45	0,45	0,44	0,45	0,45	0,52	0,52	0	0,04	8	0	4	9
S	0,02	0,02	0,03	0,03	0,03	0,03	0,04	0,05	0,05	0	0,01	30	0,5	−0,01	9
Р	0,03	0,03	0,03	0,04	0,04	0,03	0,04	0,04	0,04	0	0	10	0	−1,45	9
Mn	705	705	751	782	780	775	821	868	868	2737	52	7	0,26	−0,64	9
Pb	17	17	22,5	25	25	26	27	37	37	31	5,6	22	0,8	1,87	9
Co	17,5	17,5	18	21	21	19	24	29	29	15	3,9	19	1,29	0,92	9
Ni	57	57	59	63	63	60	69	71	71	37	6,1	10	0,22	−2,35	9
Sr	162	162	167	190	189	191	201	243	243	727	27	14	0,88	0,33	9
Cu	34	34	37	41	41	43	45	49	48,5	26	5,1	12	−0,06	−1,46	9
Zn	70	70	74	89	87	86	106	115	115	298	17	19	0,55	−1,52	9
Cr	153	153	154	164	163	161	169	187	187	138	12	7	1,01	0,46	9
V	121	121	136	150	149	152	156	190	190	390	20	13	0,7	1,34	9
Rb	81	81	85	90	90	92	93,5	97	97	36	6,0	7	−0,49	−1,21	9
Ba	421	421	463	497	495	483	537	560	560	2379	49	10	−0,08	−1,43	9
Th	6	6	8,5	40	20	11	102	102	102	2161	46	116	0,85	−1,71	9
Argic															
G1	7,8	7,9	8,1	8,9	8,8	8,7	9,4	9,9	12,0	1,0	1,0	11	1,64	3,77	20
G2	23,2	23,4	26,0	28,2	28,0	28,6	29,9	32,8	34,0	10,0	3,2	11	−0,01	−0,6	20
G3	8,7	11,3	13,9	15,1	14,9	15,9	16,8	17,6	19,3	6,6	2,6	17	−1,07	1,27	20
G4	39,2	41,4	42,5	44,7	44,6	44,9	46,2	48,7	50,7	8,5	2,9	7	0,27	0,02	20
G5	0,4	0,6	1,1	2,6	2,0	2,4	3,5	4,9	7,0	3,2	1,8	69	0,81	0,22	20
G6	0	0	0	0,5		0	0	2,4	5,8	2,1	1,5	268	3,05	9,43	20
pH	6,2	6,4	6,7	7,0	7,0	6,8	7,5	7,7	7,8	0,3	0,5	7	0,01	−1,39	20
TOC	0,23	0,75	0,87	1,08	1,01	1,06	1,31	1,58	1,82	0,13	0,36	33	−0,02	0,92	20
Fe	4,87	4,91	4,99	5,1	5,09	5,05	5,2	5,29	5,67	0,03	0,18	4	1,65	3,93	20
Si	28	28	29	29	29	29	30	30	32	0,71	0,84	3	1,76	6,29	20
Al	7,62	8,14	8,23	8,4	8,4	8,39	8,6	8,76	8,84	0,08	0,28	3	−0,76	1,68	20
Ca	0,78	0,8	0,83	0,9	0,89	0,85	0,93	1,01	1,39	0,02	0,13	15	2,92	10,06	20
Mg	1,14	1,2	1,31	1,34	1,34	1,38	1,38	1,44	1,44	0,01	0,09	6	−1,01	0,38	20
Ti	0,71	0,74	0,74	0,75	0,75	0,76	0,76	0,77	0,78	0	0,02	2	−1,11	2,14	20
Na	0,37	0,37	0,37	0,43	0,43	0,45	0,45	0,45	0,73	0,01	0,08	18	2,93	11,3	20
S	0	0	0,01	0,02		0,02	0,02	0,03	0,04	0	0,01	66	−0,29	0,11	20
Р	0,03	0,03	0,03	0,04	0,04	0,04	0,04	0,04	0,04	0	0	9	0,62	−0,8	20
Mn	775	782	802	832	831	825	856	900	906	1677	41	5	0,57	−0,66	20
Pb	15	21	27	29	28	29	32	36	39	32	5,69	20	−0,73	1,11	20
Co	11	16	18	21	20	20	25	26	28	20	4,44	21	−0,39	−0,36	20
Ni	56	65	67	69	69	70	71	76	78	25	5	7	−0,49	1,45	20
Sr	131	136	142	148	148	151	156	158	163	80	9,0	6	−0,37	−0,77	20
Cu	32	33	37	46	43	40	44	75	119	446	21	46	2,76	7,74	20
Zn	82	86	93	110	108	104	125	144	163	508	23	20	0,79	−0,07	20
Cr	183	189	193	200	200	201	206	213	214	80	8,9	4	−0,09	−0,86	20
V	171	174	179	184	184	185	189	192	195	50	7,0	4	−0,42	−0,97	20
Rb	97	99	101	104	104	103	108	113	114	26	5,1	5	0,6	−0,65	20
Ba	498	506	514	531	530	530	541	566	569	418	20	4	0,46	−0,42	20
Th	5	7	8	14	10	9	11	13	102	435	21	151	4,4	19,57	20
Mollic															
G1	5,7	6,1	7,4	7,9	7,8	7,9	8,5	9,3	9,5	1,2	1,1	14	−0,44	−0,44	22
G2	20,7	23,9	27,0	30,1	29,8	30,1	33,2	35,2	37,9	18,9	4,4	14	−0,32	−0,34	22
G3	3,2	8,4	12,5	14,1	13,4	15,3	16,1	17,6	19,4	14,3	3,8	27	−1,42	2,24	22
G4	36,4	38,3	40,0	43,5	43,2	42,3	48,0	50,8	56,6	27,4	5,2	12	0,98	0,31	22
G5	0,2	0,7	1,2	2,8	1,9	2,2	2,6	4,7	16,0	10,7	3,3	116	3,41	13,41	22
G6	0	0	0	1,5		0	0,02	5,9	18,8	19,7	4,4	291	3,33	11,67	22
pH	6,1	6,3	6,7	6,8	6,8	6,8	7,1	7,3	7,4	0,1	0,4	5	−0,15	−0,3	22
TOC	0,7	2,0	3,8	4,7	4,1	4,9	6,0	6,4	9,3	3,8	2,0	42	−0,17	0,77	22
Fe	3,6	4,2	4,5	4,6	4,6	4,6	4,8	4,9	5,6	0,16	0,4	9	−0,25	2,32	22
Si	30	32	32	33	33	33	33	34	35	1,3	1,1	3	−0,68	2,84	22
Al	6,2	7,1	7,6	7,6	7,6	7,7	7,8	8,4	8,6	0,28	0,53	7	−0,74	1,69	22
Ca	0,81	0,99	1,06	1,15	1,14	1,12	1,25	1,32	1,51	0,02	0,15	13	0,02	0,74	22
Mg	0,82	1,08	1,1	1,15	1,14	1,16	1,21	1,24	1,44	0,01	0,12	10	−0,54	2,94	22
Ti	0,75	0,76	0,77	0,78	0,78	0,77	0,8	0,81	0,82	0	0,02	3	0,42	−0,89	22
Na	0,45	0,57	0,59	0,61	0,61	0,6	0,67	0,68	0,68	0	0,07	11	−1,23	1,51	22
S	0	0,02	0,02	0,03		0,03	0,05	0,06	0,09	0	0,02	58	0,77	1,86	22
Р	0,03	0,05	0,05	0,06	0,06	0,06	0,08	0,08	0,08	0	0,01	21	−0,24	−0,64	22
Mn	829	932	1112	1145	1138	1168	1202	1243	1400	17189	131	11	−0,63	1,18	22
Pb	17	22	25	28	28	28	32	33	36	21	4,6	16	−0,35	0	22
Co	17	17	18	21	21	21	23	26	32	15	3,9	18	1,3	1,59	22
Ni	39	47	49	56	55	56	61	68	73	66,62	8,2	15	0,09	0,04	22
Sr	155	159	161	175	174	173	186	190	195	155	12	7	−0,03	−1,24	22
Cu	33	37	43	50	49	49	60	62	65	93	9,6	19	−0,07	−1,15	22
Zn	104	112	127	139	138	135	157	166	182	469	22	16	0,33	−0,46	22
Cr	153	183	207	217	215	212	232	248	289	923	30	14	0,46	1,37	22
V	141	152	171	178	177	180	187	197	208	271	16	9	−0,54	0,16	22
Rb	96	104	105	108	108	109	112	113	116	23	4,8	4	−0,87	0,83	22
Ba	505	516	539	571	570	584	596	608	636	1403	37	7	−0,27	−0,9	22
Th	5	6,73	7,94	18	12	10	12	16	102	743	27	150	3	7,85	22
Key site 3. Chernozems															
(Proto)calcic															
G1	7,3	7,3	9,6	15,1	13,3	11,3	18,1	35,4	35,4	87,0	9,3	62	1,82	3,21	8
G2	23,7	23,7	27,6	31,8	31,3	30,4	36,8	41,3	41,3	37,7	6,1	19	0,52	−0,82	8
G3	7,5	7,5	10,1	12,5	12,2	14,0	14,7	15,1	15,1	8,3	2,9	23	−0,92	−0,74	8
G4	12,4	12,4	31,3	32,4	30,8	35,0	36,7	40,4	40,4	75,1	8,7	27	−2,12	5,12	8
G5	0,0	0,0	3,0	8,1	3,8	7,2	14,1	15,7	15,7	38,8	6,2	77	0,12	−2	8
G6	0,02	0,02	0,03	0,15	0,09	0,12	0,24	0,44	0,44	0,02	0,15	97	1,08	0,37	8
pH	7,4	7,4	8,3	8,2	8,2	8,3	8,4	8,5	8,5	0,1	0,4	4	−2,55	6,89	8
TOC	0,45	0,45	0,66	1,1	0,96	0,88	1,39	2,51	2,51	0,44	0,67	60	1,49	2,41	8
Fe	4,2	4,2	4,3	4,4	4,4	4,3	4,4	4,9	4,9	0,05	0,22	5	2,43	6,39	8
Si	24	24	25	25	25	25	26	27	27	0,37	0,6	2	0,57	1,32	8
Al	6,5	6,5	6,8	6,8	6,8	6,8	6,8	6,9	6,9	0,02	0,13	2	−2,31	6,06	8
Ca	1,0	1	5,1	4,9	4,4	5,3	5,6	6,0	6,0	2,55	1,6	33	−2,58	7,01	8
Mg	1,2	1,2	1,26	1,34	1,34	1,35	1,41	1,5	1,5	0,01	0,11	8	−0,14	−0,74	8
Ti	0,62	0,62	0,62	0,63	0,63	0,63	0,63	0,67	0,67	0	0,01	2	2,19	5,23	8
Na	0,3	0,3	0,37	0,37	0,37	0,37	0,37	0,45	0,45	0	0,04	11	0	3,5	8
S	0,06	0,06	0,07	0,07	0,07	0,07	0,08	0,08	0,08	0	0,01	12	−0,28	−1,39	8
Р	0,03	0,03	0,03	0,04	0,04	0,03	0,04	0,04	0,04	0	0	9	−0,4	−0,23	8
Mn	651	651	701	727	726	717	767	798	798	2288	48	7	−0,03	−0,48	8
Pb	15	15	19,5	24	23	25	27	33	33	32	5,7	24	−0,02	−0,09	8
Co	13	13	15	17	17	18	19	20	20	7,27	2,7	16	−0,99	−0,53	8
Ni	67	67	68	71	71	70	73	84	84	32	5,7	8	1,98	3,9	8
Sr	165	165	186	202	201	211	217	222	222	417	20	10	−0,91	−0,46	8
Cu	30	30	33	41	39	37	42	80	80	263	16	39	2,43	6,25	8
Zn	66	66	69	73	73	71	73	95	95	83	9,1	12	2,42	6,34	8
Cr	165	165	168	175	174	174	181	186	186	63	7,95	5	0,15	−1,81	8
V	119	119	122	134	133	133	136	168	168	243	16	12	1,72	3,7	8
Rb	358	358	384	407	405	404	414	494	494	1608	40	10	1,56	3,66	8
Ba	79	79	79	81	81	80	82	90	90	14	3,7	5	2,39	6,05	8
Th	6	6	7	31	15	9	56	102	102	1901	44	139	1,44	0	8
Chernic															
G1	4,6	5,3	6,4	6,8	6,8	7,0	7,4	8,0	8,1	0,9	1,0	14	−1,06	1,31	14
G2	19,5	21,3	26,8	28,0	27,8	28,7	30,5	31,2	32,2	13,1	3,6	13	−1,47	1,81	14
G3	7,3	9,2	11,4	14,6	14,1	16,1	16,8	18,2	19,4	13,0	3,6	25	−0,79	−0,35	14
G4	28,2	31,4	38,2	39,1	38,8	39,7	42,1	43,9	45,5	22,4	4,7	12	−1,11	1,08	14
G5	1,5	2,9	5,4	7,4	6,5	6,7	10,0	10,7	15,3	12,5	3,5	48	0,54	0,78	14
G6	0,01	0,02	0,05	4,1	0,17	0,09	0,24	26,6	30,0	105,3	10,3	248	2,31	3,94	14
pH	6,4	6,5	6,6	6,8	6,8	6,7	6,9	7,6	7,6	0,13	0,36	5	1,65	2,08	14
TOC	2,9	5,0	6,3	6,9	6,7	7,0	7,4	8,5	10,2	2,83	1,68	24	−0,56	2,06	14
Fe	3,3	3,4	3,4	3,6	3,6	3,6	3,7	4,0	4,2	0,06	0,24	7	1,22	1,42	14
Si	23	23	23	24	24	24	25	25	26	0,86	0,93	4	1,04	1,05	14
Al	5,3	5,3	5,5	5,8	5,8	5,7	5,9	6,4	6,7	0,16	0,4	7	1,03	0,71	14
Ca	0,89	0,99	1,01	1,05	1,05	1,03	1,12	1,14	1,15	0,01	0,07	7	−0,42	0,22	14
Mg	0,81	0,81	0,82	0,91	0,91	0,89	0,99	1,01	1,2	0,01	0,11	12	1,3	2,02	14
Ti	0,47	0,49	0,5	0,52	0,52	0,51	0,54	0,57	0,59	0	0,03	6	1	0,72	14
Na	0,38	0,44	0,44	0,45	0,45	0,44	0,45	0,47	0,51	0	0,03	6	−0,11	4,25	14
S	0,02	0,02	0,03	0,04	0,03	0,03	0,04	0,06	0,06	0	0,01	41	0,53	−0,72	14
Р	0,04	0,05	0,06	0,06	0,06	0,06	0,06	0,07	0,07	0	0,01	13	−0,66	0,44	14
Mn	720	724	748	770	769	766	800	819	835	1268	36	5	0,37	−0,73	14
Pb	13	16	18	21	21	19	24	31	33	31	5,6	26	0,84	0,22	14
Co	11	13	13	16	16	16	18	20	23	10	3,2	20	0,51	−0,24	14
Ni	38	39	41	45	45	45	48	52	57	29	5,3	12	0,58	0,25	14
Sr	139	143	147	148	148	149	151	153	153	17	4,1	3	−1,08	0,91	14
Cu	24	24	28	30	30	30	31	34	38	15	3,9	13	0,25	0,84	14
Zn	89	93	100	114	113	116	129	132	138	232	15	13	−0,26	−0,88	14
Cr	132	139	150	158	158	157	168	184	185	253	16	10	0,24	−0,46	14
V	121	124	126	134	134	130	143	149	150	98	10	7	0,45	−1,21	14
Rb	366	388	397	413	412	411	435	441	451	553	24	6	−0,19	−0,4	14
Ba	74	75	76	77	77	77	79	80	82	4,2	2,0	3	0,51	−0,02	14
Th	0	0	4,4	6,5	na	6,4	10	11	12	14	3,8	58	−0,28	−0,53	14
Mollic-argic															
G1	7,1	7,1	7,1	10,1	9,7	9,6	11,3	16,1	16,1	11,3	3,4	33	1,25	1,72	6
G2	25,9	25,9	26,2	30,5	30,1	28,5	33,8	40,2	40,2	32,1	5,7	19	1,17	0,58	6
G3	10	10	12	15	14	16	17	18	18	9,3	3,0	21	−0,91	−1,03	6
G4	29	29	36	37	37	38	39	43	43	21	4,6	12	−1,14	2,31	6
G5	2,5	2,5	2,6	7,2	6,0	7,9	10,9	11,5	11,5	17,8	4,2	59	−0,19	−2,74	6
G6	0	0	0,02	0,1	na	0,05	0,11	0,38	0,38	0,02	0,14	142	2,1	4,57	6
pH	7,0	7,0	7,5	7,6	7,6	7,7	7,7	8,1	8,1	0,1	0,3	4	−0,65	1,84	6
TOC	0,9	0,9	1,1	2,3	2,0	2,3	3,3	3,7	3,7	1,4	1,2	51	−0,05	−1,79	6
Fe	4,1	4,1	4,1	4,4	4,4	4,2	4,9	5,0	5,0	0,2	0,4	10	0,93	−1,7	6
Si	27	27	27	27	27	27	28	28	28	0,33	0,58	2	0,9	−0,15	6
Al	6,5	6,5	6,5	6,8	6,8	6,5	7,2	7,7	7,7	0,26	0,51	7	1,29	0,21	6
Ca	0,82	0,82	0,87	0,94	0,93	0,91	0,95	1,18	1,18	0,02	0,13	13	1,64	3,06	6
Mg	1,03	1,03	1,04	1,15	1,14	1,09	1,26	1,38	1,38	0,02	0,14	12	1,11	−0,17	6
Ti	0,57	0,57	0,58	0,62	0,62	0,59	0,69	0,69	0,69	0	0,06	9	0,82	−1,92	6
Na	0,37	0,37	0,37	0,46	0,46	0,47	0,54	0,54	0,54	0,01	0,08	17	−0,31	−1,87	6
S	0,02	0,02	0,03	0,05	0,04	0,04	0,06	0,08	0,08	0	0,03	54	0,23	−2,12	6
Р	0,03	0,03	0,03	0,03	0,03	0,03	0,04	0,04	0,04	0	0	8	0,71	0,69	6
Mn	669	669	724	763	760	744	844	852	852	5131	72	9	0,29	−1,26	6
Pb	22	22	23	26	25	24	27	35	35	24	4,9	19	1,76	3,3	6
Co	14	14	15	19	19	20	23	24	24	18	4,3	22	−0,34	−2,37	6
Ni	49	49	51	63	62	54	84	88	88	312	18	28	0,95	−1,74	6
Sr	146	146	148	157	156	152	170	172	172	128	11	7	0,8	−1,8	6
Cu	24	24	28	31	31	30	33	43	43	42	6,5	21	1,14	2,39	6
Zn	83	83	88	110	108	113	116	147	147	535	23	21	0,5	0,39	6
Cr	166	166	187	197	196	199	214	217	217	386	20	10	−0,65	−0,76	6
V	157	157	158	164	164	160	171	177	177	67	8,2	5	1,05	−0,62	6
Rb	443	443	459	473	473	471	489	507	507	515	23	5	0,25	−0,29	6
Ba	76	76	78	84	83	81	93	93	93	57	7,6	9	0,69	−1,87	6
Th	4,6	4,6	7,3	8,7	8,3	9,1	10	12	12	6,4	2,5	29	−0,61	0,91	6

Concentration of grain fractions (G1 – clay (particles <1 μm), G2 – fine silt (1–5), G3 – medium silt (5–10), G4 – coarse silt (10–50), G5 – fine sand (50–250) and G6 – medium and coarse (250–1000) sand), TOC, Fe, Si, Al, Ca, Mg, Ti, Na, P is in %. Concentration of all other elements is in mgkg^−1^. Min – minimum, P_10_, P_25_, P_50_, P_75_, P_90_ – percentiles, M − mean, GM – geometric mean, Max – maximum, V – variance, SD – standard deviation, Cv – coefficient of variation.

Total concentration of HMs in the soils were compared to several SQSs (Table 6), because the specified countries have the similar set of soil reference groups, background concentration of HMs (Table 7) and well-developed methodologies for soil quality assessment.

**Table 6:** SQSs for Ba, Cr, Cu, Mn, Ni, Pb, V, and Zn of the five countries analysed, mg kg^−1^.

Metals	Threshold levels of soil quality standards						
	Russia [10], [11]		Netherlands, ISRCs [13]	Germany, TVs [14]	Canada, SQGs [15]		The USA, RSLs [16]
	MPCs	EPCs		IIа	I	II	IIа
Ba	–		890	–	750	500	1500
Cr	–		–	400	64		–
Cu	–	66	96	–	63		310
Mn	1500	–	–	–	–		180
Ni	–	40	100	140	70	140	–
Pb	32	65	580	400	45		–
V	150	–	–	–	130		–
Zn	–	110	350	–	250		–

MPCs – maximum permissible concentrations, EPCs – estimated permissible concentrations, ISRC – integrated serious risk concentration, TV – Trigger Values, SQG – soil quality guidelines, RSL – regional screening levels. I – agricultural lands; II – residential and parkland, IIа – residential zones.

**Table 7:** Heavy metal concentration (mg kg^−1^) in the upper part of continental earth's crust (UCEC) and background soils of the world.

Metals	UCEC or Clark [2]	World's soils [3]	Northern Europe [3]	North America [4]	Australia [3]	China [5]	Finland [6]	Western Siberia [7], [8]
Ba	510–1070	315–500	43	968	67	440–469	–	373–1360
Cr	35–92	42–200	13	130	24	37–61	31	5–190
Cu	14–47	11–13	9.4	35	18	17–25	22	5–100
Mn	527–1000	310–1007	280	538	281	333–600	–	50–1800
Ni	19–58	15–50	8.2	88	9.8	13–27	17	7–100
Pb	16–20	10–35	10	19	7.2	16–27	5	5–35
V	53–121	55–100	25	112	32	58–82	38	5–140
Zn	51–83	31–90	30	106	86.3	48–74	31	10–120

Spearman correlation analyses among the HMs and soil parameters (grain size fractions, TOC and pHwater) were conducted in order to visualize the kind of relationships that exist among the variables (Table 8).

**Table 8:** Correlation matrices between metal concentration and particle-size fractions, pHwater and TOC.

Properties	Ba	Cr	Cu	Mn	Ni	Pb	V	Zn
Retisols, n = 65								
G1, n = 49	−0.04	0.40**	0.29*	−0.11	0.64***	0.03	0.36*	0.35*
G2, n = 49	−0.13	0.19	0.06	0.14	0.37**	−0.05	0.10	0.18
G3, n = 49	−0.20	−0.01	−0.20	0.34*	0.13	−0.17	−0.11	0.05
G4, n = 49	−0.17	0.21	0.22	0.00	0.32*	−0.15	0.10	0.23
G5, n = 49	0.21	0.13	0.08	−0.40**	−0.21	0.20	0.10	−0.26
G6, n = 49	0.07	−0.34*	−0.31*	0.14	−0.06	0.28	0.05	0.15
pHwater	−0.01	−0.43***	−0.38**	0.40**	−0.09	0.17	−0.47***	0.29*
TOC	−0.29*	−0.71***	−0.70***	0.56***	−0.50***	−0.12	−0.78***	0.04
Phaeozems, n = 51								
G1	−0.16	−0.27	−0.10	−0.43**	0.20	−0.01	−0.22	−0.24
G2	0.17	0.27	0.20	0.18	−0.23	−0.07	−0.22	0.29*
G3	0.01	0.27	0.00	0.12	0.21	0.08	0.28*	0.18
G4	0.20	−0.03	−0.01	0.12	0.04	0.03	0.33*	−0.01
G5	−0.07	0.20	0.11	0.04	0.07	0.08	0.14	0.00
G6	−0.22	−0.24	−0.16	−0.25	0.07	0.11	−0.07	−0.36*
pHwater	−0.30*	−0.41**	−0.25	−0.52***	0.10	−0.13	−0.24	−0.31*
TOC	0.49***	0.59***	0.49***	0.68***	−0.45***	0.11	0.16	0.62***
Chernozems, n = 28								
G1	0.10	0.55**	0.53**	−0.07	0.78***	0.18	0.18	−0.64***
G2	−0.04	0.06	0.51**	0.19	0.31	−0.09	0.10	−0.22
G3	0.22	−0.13	−0.50**	0.11	−0.14	−0.02	0.23	0.10
G4	0.18	−0.28	−0.17	0.21	−0.38*	−0.10	0.28	0.46*
G5	−0.06	−0.20	−0.13	−0.37	−0.19	−0.05	−0.13	0.14
G6	−0.20	−0.09	0.07	−0.02	−0.15	0.13	−0.17	0.12
pHwater	0.02	0.57**	0.32	−0.31	0.77***	0.29	0.10	−0.68***
TOC	−0.14	−0.68***	−0.31	0.26	−0.81***	−0.30	−0.20	0.61***

Spearman's rank correlation coefficients marked are statistically significant at p < 0.05 (*), 0.01 (**) and 0.001 (***).

## 2 Experimental design, materials, and methods

Plastic and steel tools were used for sampling. After air-drying and declumping the aggregates, the soil was sieved through a 1 mm mesh sieve. Total organic carbon (dichromate digestion based on Walkley–Black method) and pHwater (the soil:solution ratio of 1:2.5) were determined. The particle-size distribution was analysed using a laser diffraction technique and an ‘Analizeter 22’ equipment (Germany) in samples pre-treated with 4% Na_4_P_2_O_7_ at the Lomonosov Moscow State University.

The total content of heavy metals was measured using an X-Ray fluorescence technique (Table 4) and a PANalytical spectrometer (Netherlands) at the Institute of Geology of Ore Deposits, Petrography, Mineralogy and Geochemistry of the Russian Academy of Sciences. The instrument was calibrated for trace elements in soils using a certified, standard reference material of Russian soils (SP-1 ‘Retisols’, and SP-2 ‘Chernozems’).
